# Suppression of Structural Phase Transition in VO_2_ by Epitaxial Strain in Vicinity of Metal-insulator Transition

**DOI:** 10.1038/srep23119

**Published:** 2016-03-15

**Authors:** Mengmeng Yang, Yuanjun Yang, Bin Hong, Liangxin Wang, Kai Hu, Yongqi Dong, Han Xu, Haoliang Huang, Jiangtao Zhao, Haiping Chen, Li Song, Huanxin Ju, Junfa Zhu, Jun Bao, Xiaoguang Li, Yueliang Gu, Tieying Yang, Xingyu Gao, Zhenlin Luo, Chen Gao

**Affiliations:** 1 National Synchrotron Radiation Laboratory, University of Science and Technology of China, Hefei, Anhui 230026, China; 2 Collaborative Innovation Center of Chemistry for Energy Materials, University of Science and Technology of China, Hefei, Anhui 230026, China; 3 CAS Key Laboratory of Materials for Energy Conversion, Department of Materials Science and Engineering, University of Science and Technology of China, Hefei, Anhui 230026, China; 4 Department of Physics, University of Science and Technology of China, Hefei, Anhui 230026, China; 5 Shanghai Synchrotron Radiation Facility, Shanghai Institute of Applied Physics, Chinese Academy of Sciences, Shanghai 201204, China

## Abstract

Mechanism of metal-insulator transition (MIT) in strained VO_2_ thin films is very complicated and incompletely understood despite three scenarios with potential explanations including electronic correlation (Mott mechanism), structural transformation (Peierls theory) and collaborative Mott-Peierls transition. Herein, we have decoupled coactions of structural and electronic phase transitions across the MIT by implementing epitaxial strain on 13-nm-thick (001)-VO_2_ films in comparison to thicker films. The structural evolution during MIT characterized by temperature-dependent synchrotron radiation high-resolution X-ray diffraction reciprocal space mapping and Raman spectroscopy suggested that the structural phase transition in the temperature range of vicinity of the MIT is suppressed by epitaxial strain. Furthermore, temperature-dependent Ultraviolet Photoelectron Spectroscopy (UPS) revealed the changes in electron occupancy near the Fermi energy *E*_F_ of V 3d orbital, implying that the electronic transition triggers the MIT in the strained films. Thus the MIT in the bi-axially strained VO_2_ thin films should be only driven by electronic transition without assistance of structural phase transition. Density functional theoretical calculations further confirmed that the tetragonal phase across the MIT can be both in insulating and metallic states in the strained (001)-VO_2_/TiO_2_ thin films. This work offers a better understanding of the mechanism of MIT in the strained VO_2_ films.

Vanadium dioxide (VO_2_) is an archetypal correlated material discovered by Morin with excellent metal-insulator transition (MIT) characteristics at the critical temperature (~68 °C in bulk state)^1^. Due to the optical transmittance changes at the infrared^2^ and THz regions^3^,^4^ and huge resistance jump^5^,VO_2_ has become a widely-studied material in fundamental studies and for industrial applications such as metamaterials^6^,^7^,^8^, smart windows^9^, supercapacitors^10^, etc. Generally, the MIT of bulk VO_2_ or nanobeam-like counterparts always accompanies a structural phase transition from a low temperature monoclinic phase to a high temperature tetragonal phase^11^,^12^,^13^. However, this hypothesis does not always hold.

Indeed, there is still a significant debate over whether the mechanism of MIT in VO_2_ is a Mott transition^14^,^15^, Peierls transition^16^,^17^, or a combination of the two^18^,^19^. As early as 1975, Zylbersztejn and Mott showed that the mechanism of MIT in VO_2_ was not the simple Mott-Hubbard transition induced only by electron-electron interactions^20^. In 2004, Cavalleri *et al*. found evidence for a structure-driven MIT in VO_2_ by ultrafast spectroscopy and declared that the mechanism of MIT was not the Mott-Hubbard transition^11^. However, Kim *et al*. observed that the tetragonal metallic phase did not occur simultaneously with MIT by the ultrafast pump-probe technology—they ascribed the MIT to the Mott mechanism illustrated by photo-assisted hole excitation^21^. Thus, the mechanism of MIT remains controversial—some researchers^22^,^23^ believe that the MIT is induced by the broken symmetry of lattice, namely, the Peierls transition. Concurrently, there are many others who believe that the MIT in VO_2_ is a Mott transition^14^,^24^.

On the other hand, epitaxial strain due to lattice mismatch between thin film VO_2_ and substrates has revealed a different picture of the MIT^22^. A tetragonal phase was identified by transmission electron microscopy in the ultrathin VO_2_ layers just adjacent to the TiO_2_ substrate in Zou’s work^25^, and X-ray diffraction reciprocal space mapping (XRD-RSM) suggested that there is no monoclinic phase in ultrathin VO_2_ films at room temperature^25^. Furthermore, our recent findings demonstrated an anomalous tetragonal-like to tetragonal structural phase transition but not a conventional monoclinic to tetragonal phase transition in 300-nm-thick VO_2_/TiO_2_ films (not shown here). In addition, Jiwei Lu *et al*. proposed a Mott-like phase transition in VO_2_ thin films induced by a large bi-axial epitaxial strain based on Raman spectroscopy data^26^. Laverock *et al*. observed a crossover from a Mott-Peierls-like transition to a Mott-like transition by large out-of-plane tensile in strained VO_2_ thin films through various spectroscopic techniques^27^.

All of these observations have demonstrated that the structural evolution in the strained VO_2_ thin films was complicated by epitaxial strain, which could lead a misunderstanding of the role of structural phase transition in the MIT. Therefore, epitaxial strain plays a very important part in not only modulating the MIT behaviors but also understanding the underlying mechanism of MIT in the strained VO_2_ thin films. To date, the mechanism of MIT in strained VO_2_ thin films has not been completely addressed. Moreover, the low-temperature phase in thin film VO_2_ under substrate clamping has not yet been fully understood and identified—this is very important for unveiling the MIT mechanism in ultrathin VO_2_ epitaxial films.

In this study, we investigated the evolutions of crystal structure and electronic states in the ultrathin VO_2_ films grown on (001)-oriented TiO_2_ substrates via temperature-dependent XRD-RSM, Raman spectroscopy and Ultraviolet Photoelectron Spectroscopy (UPS). We found that the absence of the structural phase transition across the MIT—and thus the electronic transition—may solely trigger the MIT. Moreover, the insulating tetragonal VO_2_ at room temperature is stabilized by epitaxial strain, which is further confirmed by density functional theory (DFT) calculations. To the best of our knowledge, this is a systematic report to investigate the structural and electronic transition of VO_2_ films grown on (001)-oriented TiO_2_ substrates combining XRD-RSM, Raman spectroscopy, UPS tools and theoretical methods—the findings also have important implications for researchers in the field of optics and electronics.

## Results and Discussion

### Crystal structure evolution across MIT

**Figure 1:**
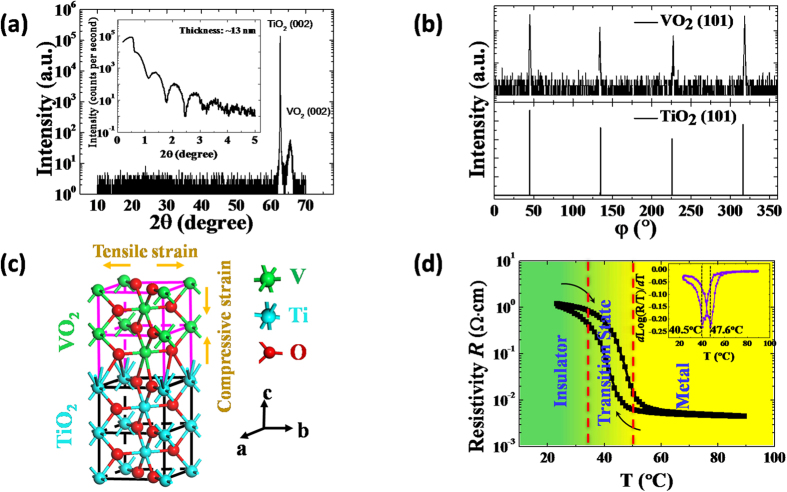
(**a**) XRD *θ*–2*θ* patterns of ultrathin VO_2_/TiO_2_ film, inset: XRR curve. (**b**) The XRD *φ* scans of the (101) peaks of VO_2_ film and TiO_2_ substrate at room temperature. (**c**) The schematic crystal structures of ultrathin VO_2_ film on TiO_2_ substrate. (**d**) The R-T curve of the ultrathin VO_2_/TiO_2_ film and corresponding differential curve.

To evaluate the quality of the VO_2_ thin film, the XRD *θ*–2*θ* patterns of (001)-VO_2_/TiO_2_ thin film were acquired (Fig. 1a). As shown in Fig. 1a, only the (002) peaks of the VO_2_ thin film and TiO_2_ substrate appear, which suggests that the VO_2_ thin film is highly oriented along the out-of-plane direction of the TiO_2_ substrate. According to the small angle X-ray reflection (XRR) in the inset of Fig. 1a, there are clear thickness-interference fringes suggesting that the surface of the VO_2_ thin film is smooth. Both atomic force microscopy and polarized optical microscopy confirmed the smooth surface (root mean square: ~0.4 nm) and uniformity of VO_2_ thin films (not shown here). We thus can calculate the thickness (*h*) with the formula^28^:


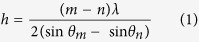


where *m* and *n* are the orders of interference, and *θ*_*m*_ and *θ*_*n*_ are the corresponding diffraction angles, respectively. The VO_2_ thickness is calculated to be ~13 nm, which is thin enough to maintain the epitaxial strain between the VO_2_ thin film and TiO_2_ substrate well below the critical thickness (~26.5 nm)^26^,^29^.

From the XRD *φ* scans of (101) peaks of TiO_2_ substrate at room temperature in Fig. 1b, there are four peaks, each separated by 90^o^. This originates from the nature of four-fold symmetry of the TiO_2_ substrate. For the VO_2_ thin film, the (101) peaks (defined by tetragonal unit cell) from the *φ* scans have located the same azimuthal angles as those in the TiO_2_ substrate (Fig. 1b). Therefore, the 13-nm VO_2_ thin film grows epitaxially on the tetragonal TiO_2_ substrate and has a similar symmetry as the TiO_2_ substrate at room temperature. This implies the four-fold symmetry of the VO_2_ thin film. And thus the epitaxial relationship between the VO_2_ thin film and TiO_2_ substrate is 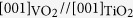 along the out-of-plane direction, and 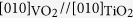 and 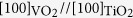 along the in-plane direction. This relationship is shown schematically at Fig. 1c. We can imagine that the ultrathin VO_2_ film probably suffers tensile strain along the in-plane direction because the in-plane lattice constant *a* of TiO_2_ substrate is larger than that of bulk VO_2_. The compressive strain should be correspondingly developed along the out-of-plane direction with assumption of conservation of volume of the VO_2_. The strain states in the (001)-VO_2_ epitaxial film are shown in Fig. 1c.

Importantly, the 13-nm (001)-VO_2_ epitaxial film is in the tetragonal phase at room temperature as shown by the above symmetry analysis of the diffraction peaks. The Raman data offers additional evidence regarding the low-temperature tetragonal phase in this VO_2_ thin film, but this contradicts with the previous reports^30^ in which the monoclinic phase (M1) of the 110 nm-thick VO_2_ films were studied at room temperature. We conjecture that epitaxial strain should stabilize the tetragonal phase in the thinner (001)-VO_2_/TiO_2_ film versus the cases in the literature^27^,^31^ at room temperature. However, the relaxation of the epitaxial strain in the thicker VO_2_ thin film resulted in the formation of the observed M1 phase at room temperature (see Raman spectra below).

Figure 1d shows the *R*-*T* curve of the ultrathin (001)-VO_2_/TiO_2_ film and corresponding differential curve during the temperature cycling from room temperature to 90 °C and backward. The resistivity shows an abrupt jump at the transition temperature (T_MIT_) of ~47.6 °C (Fig. 1d inset), which is lower than that of the bulk counterpart. Moreover, the change of resistivity across the MIT is up to three orders of magnitude, which is comparable to previous reports^25^. These could be closely related to the modulation of the electronic structures by epitaxial strain^32^,^33^,^34^. It is mentionable that the MIT in the present work is a bit higher than the previous reports (~36 °C)^26^ with the similar film thickness. First, the Ti interdiffusion partially accounts for the increasing the MIT in the VO_2_/TiO_2_ epitaxial films. Y. Muraoka *et al*. found that the high growth temperature can induce the Ti diffusion to the VO_2_ thin films and dramatically increase the MIT from 28 °C to 67 °C in the 12-nm (001)-VO_2_/TiO_2_ films grown by pulse laser deposition technique^35^. And the interdiffusion region was observed to be restricted to 1.2 nm at the interface between the VO_2_ film and TiO_2_ substrate by Quackenbush *et al*.^36^. Second, the oxygen pressure for the VO_2_ film growth may introduce non-stoichiometry and change the MIT obviously. Stuart S. P. Parkin *et al*.^37^ found the MIT of the 10-nm (001)- VO_2_/TiO_2_ films was changed from 17 °C to 28 °C with increasing the growth oxygen pressure. Junhao Chu *et al*.^38^ also observed a dramatic increasing of the MIT from 48 °C to 75 °C in the VO_2_/Al_2_O_3_ epitaxial films. The possible reason for this may be ascribed to the balance of the doping level and acceptor level in the forbidden gap produced by oxygen vacancy and vanadium vacancy in different growth oxygen pressure, respectively^38^,^39^. Third, the MIT becomes higher with the larger lattice constant *c* as reported in refs 26,35,37. The long V^4+^–V^4+^ distance relieves the direct overlapping of d orbitals, which decreases the width of the d band and increases the MIT in the tetragonal structure of the (001)-VO_2_/TiO_2_ films^35^. Therefore, the structural studies and electrical measurements both highlight the excellent quality of the sample and these samples demonstrate the apparent MIT in the (001)-VO_2_/TiO_2_ epitaxial films, which is as similar as the metal-insulator transition behaviors in the previous studies^34^,^35^,^36^,^37^,^38^,^39^.

**Figure 2:**
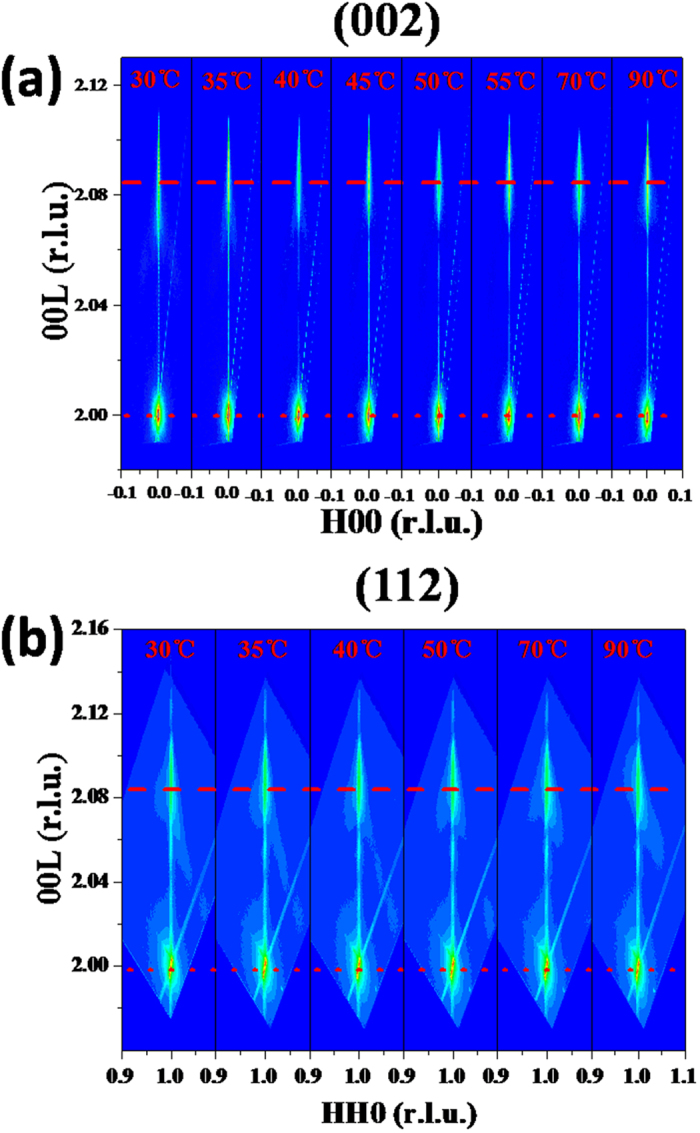
Temperature-dependent RSMs of (**a**) (002) and (**b**) (112) diffraction spots for ultrathin (001)-VO_2_/TiO_2_ thin film.The red dotted and dashed lines represent the positions of the TiO_2_ substrate and VO_2_ film, respectively.

To illustrate the correlations between the mechanism of MIT and structural phase transition in the strained (001)-VO_2_/TiO_2_ epitaxial film, the structure evolution across MIT was studied by *in situ* temperature-dependent high resolution XRD with reciprocal space mapping (RSM) technique. The evolution of RSMs around (002) and (112) diffractions of VO_2_/TiO_2_ are presented in Fig. 2. There are two Bragg spots in each RSM—the lower one belongs to TiO_2_ substrate and the upper one belongs to the VO_2_ film. The RSMs were plotted in reciprocal lattice units (r.l.u.) of the TiO_2_ substrate (1 r.l.u. = 2π/2.9592 Å^−1^). The (002) and (112) peaks of the VO_2_ film in the RSMs (Fig. 2) didn’t move across all the entire heating and cooling process. This strongly indicates that there is no structural phase transition occurring during the MIT. In other words, this behaviour is not consistent with the previous reports in the refs.^11^, ^12^, ^13^, in which the electronic transition is always accompanied with a structural phase transition. However, Jiwei Lu *et al*.^26^ recently reported a Mott-like phase transition in VO_2_ films induced by a large epitaxial bi-axial strain using Raman spectroscopy. This suggests that the structural phase transition did not always appear during the MIT. Our findings in the strained (001)-VO_2_/TiO_2_ films agree well with the Lu’s results. Moreover, this study offers direct evidence of a microscopic crystal structure for the suppression of structural phase transition by epitaxial strain. Therefore, we believe that MIT in this ultrathin (001)-VO_2_/TiO_2_ film may be driven solely by electronic transition.

From the RSMs of (002) and (112) peaks of the ultrathin (001)-VO_2_/TiO_2_ film, the lattice constants *c* and *a* allow calculation of strain:


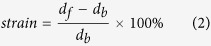


where *d*_*f*_ and *d*_*b*_ are the lattice constants of the VO_2_ thin film and its bulk counterpart, respectively. The corresponding strains were calculated to be −0.58% along the out-of-plane direction and +0.86% along the in-plane direction without relaxation of the in-plane strain—this is consistent with the previous report^40^. Thus, the ultrathin (001)-VO_2_/TiO_2_ films suffer the bi-axial strains, suppressing the structural phase transition across the MIT.

**Figure 3:**
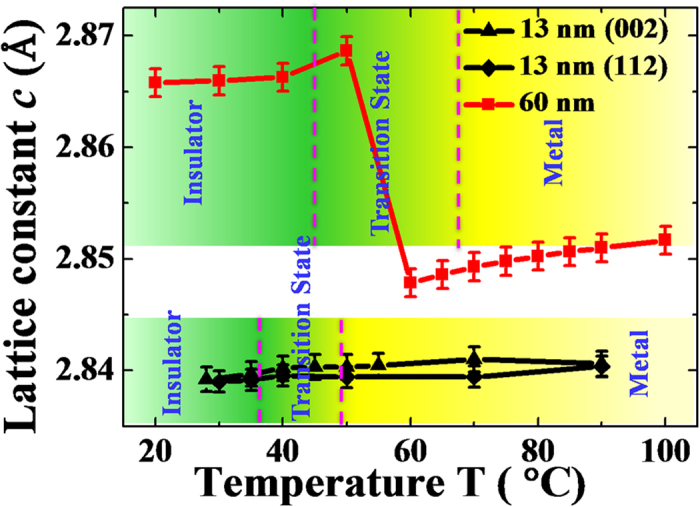
The lattice constant *c* as a function of temperature for 13-nm and 60-nm VO_2_ films. The red square line represents the *c* of 60-nm VO_2_ thin film. The triangle and diamond lines represent the *c* of the 13-nm VO_2_ thin film calculated from (002) and (112) diffraction spots in the RSMs, respectively. The mixed regions of yellow and green shadows highlight the transition temperature ranges of the 13-nm and 60-nm films that were divided by dashed lines.

To directly see the changes in the lattice constant across the MIT, we calculated the out-of-plane lattice constants based on the temperature-dependent RSMs in Fig. 2 and plotted them in Fig. 3. For comparison, we prepared a thicker (001)-VO_2_/TiO_2_ film (~60 nm) and the corresponding out-of-plane lattice constants *c* is shown in the upper part of Fig. 3. The thicker film presented an obvious abrupt jump in the lattice constant *c*, which clearly suggests a structural phase transition during MIT. Moreover, the lattice constant *c* is about 2.854 Å in the high-temperature tetragonal phase (above 65 °C), which is nearly as same as the bulk one (~2.8557 Å). Therefore, the epitaxial strain should be also relaxed along the in-plane directions in the thicker films. The temperature-dependent XRD *θ*–2*θ* fine scans to determine lattice constant *c* are shown in Supporting Information (Figs S1 and S2). However, the lattice constants *c* of the 13-nm-thick VO_2_/TiO_2_ epitaxial film were nearly constant regardless of calculation via the (002) or (112) diffraction spots. The slight increase in the lattice *c* may be due to the thermal expansion of the VO_2_^36^. This result directly shows that there is no structural phase transition during MIT for the strained (001)-VO_2_/TiO_2_ epitaxial film.

### Raman analysis for the microstructure across the MIT

**Figure 4:**
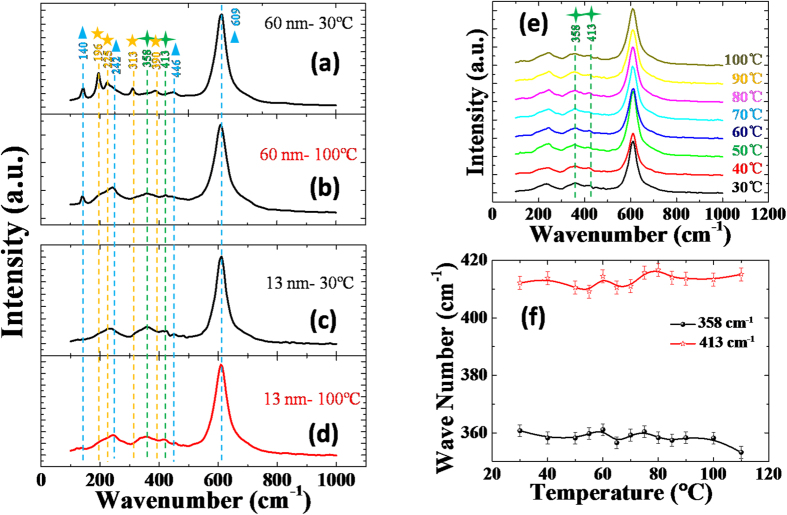
Raman spectrum of 60 nm (001)-VO_2_/TiO_2_ thin film at (**a**) 30 °C and (**b**) 100 °C of the 13-nm (001)-VO_2_/TiO_2_ thin film at (**c**) 30 °C and (**d**) 100 °C. The blue triangles, yellow stars, and green squares and corresponding dotted lines label the Raman peaks in the TiO_2_ substrate, the monoclinic phase VO_2_, and the tetragonal phase VO_2_, respectively. (**e**) Temperature-dependent Raman spectroscopy of ultrathin (001)-VO_2_/TiO_2_ thin film. (**f**) The Raman peaks belong to the tetragonal phase VO_2_ and are a function of temperature.

To further confirm the lack of structural phase transition in the ultrathin VO_2_ thin film, Raman scattering spectroscopy was used to study the lattice vibrations of VO_2_ across the MIT^41^,^42^,^43^,^44^,^45^,^46^. It is well known that the monoclinic and tetragonal phases of VO_2_ thin films have the distinctly different Raman features: the former displays several sharp Raman peaks, while the latter is characterized by a broadband peak^41^. Hence we performed temperature-dependent Raman spectroscopy measurements for both thick and thin VO_2_ films as shown in Fig. 4.

The data in Fig. 4a shows the Raman spectrum of the 60 nm-thick VO_2_/TiO_2_ thin film at 30 °C with four sharp peaks at 196 cm^−1^, 225 cm^−1^, 313 cm^−1^, and 390 cm^−1^. These peaks are the representative A_g_ vibration modes of the monoclinic VO_2_^37^,^38^,^39^,^40^. However, these four characteristic Raman peaks disappears at 100 °C (Fig. 4b). This indicates that the 60-nm-thick VO_2_/TiO_2_ film undergoes a structural phase transition from low-temperature monoclinic phase to high temperature tetragonal phase during the MIT. On the other hand, the above four Raman peaks belonging to monoclinic phase VO_2_ do not exist in the Raman spectrum of the 13-nm-thick VO_2_ thin film at 30 °C or 100 °C (see Fig. 4c,d). Hence, there are no monoclinic components of the VO_2_ in the strained (001)-VO_2_/TiO_2_ thin film at low temperature or high temperature. Furthermore, the Raman spectrum of the ultrathin VO_2_ thin film at 30 °C and 100 °C both have the two broad Raman peaks at about 358 cm^−1^ and 413 cm^−1^, which resembles the Raman spectrum of the thicker VO_2_ thin film at 100 °C in Fig. 4b, where the VO_2_ is exactly right in the tetragonal symmetry. Therefore, it is suspected that the above two broad Raman peaks belong to the tetragonal phase VO_2_ based on the conclusion that 60-nm-thick VO_2_ films experience a MIT from low temperature monoclinic phase to high temperature tetragonal phase. Interestingly, the two peaks did not move across the MIT for the case of the ultrathin (001)-VO_2_/TiO_2_ thin film in Fig. 4c,d, suggesting that the tetragonal phase in the ultrathin (001)-VO_2_/TiO_2_ film is stabilized by epitaxial strain regardless of low or high temperature.

In order to clearly show the microstructure evolution of the ultrathin (001)-VO_2_/TiO_2_ film across the MIT, we preformed the fine temperature-dependent Raman spectroscopy measurements as shown in Fig. 4e (Supporting Information Fig. S3). There are no sharp Raman peaks belonging to the monoclinic phase during the MIT indicating the absence of monoclinic phase in the strained (001)-VO_2_ thin films. In addition to the Raman peaks of the TiO_2_ substrate (140 cm^−1^, 242 cm^−1^, 446 cm^−1^, and 609 cm^−1^; blue triangles in Fig. 4d, Supporting Information Fig. S4)^45^, there are only two Raman peaks (358 cm^−1^ and 413 cm^−1^)—these belong to the tetragonal phase VO_2_. For clarity, we plotted the two peak positions as a function of temperature in Fig. 4f. The two peaks showed no clear shift during MIT, implying that there is no structural phase transition across the MIT for the strained (001)-VO_2_/TiO_2_ thin films.

Based on these XRD studies and Raman spectroscopy analysis, we concluded that the tetragonal phase is stabilized by epitaxial strain—there is no monoclinic VO_2_ at room temperature, but instead of the tetragonal phase. Consequently, the structural phase transition is not a necessary condition for the MIT in strained (001)-VO_2_/TiO_2_ thin films.

### Studies on electron occupancy near Fermi level by temperature-dependent UPS

**Figure 5:**
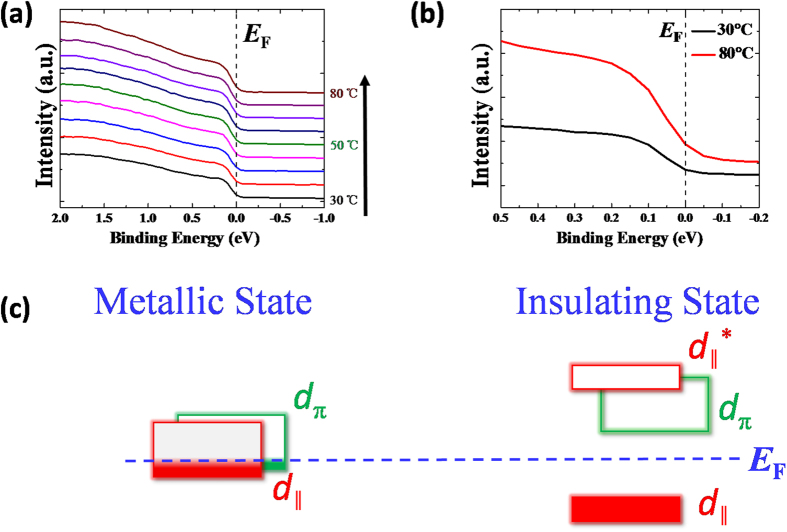
(**a**) The V 3d orbital electronic states near Fermi energy *E*_F_ with varying temperature from 30 to 80 °C. (**b**) The selected and expanded V 3d electronic states at 30 °C and 80 °C. (**c**) The band occupancies of V 3d electrons near Fermi energy *E*_F_ both at insulating and metallic states.

We next mainly studied the electronic transition of the ultrathin (001)-VO_2_/TiO_2_ thin film across the MIT by temperature-dependent UPS^47^,^48^,^49^,^50^,^51^,^52^ because the structural phase transition was suppressed in the vicinity of the MIT temperature in such strained films. Figure 5a shows the UPS of ultrathin (001)-VO_2_/TiO_2_ thin film from 30 °C to 80 °C to track the change of the V 3d electronic states from low temperature insulating state to high temperature metallic states. For clarity, the UPS spectrum at 30 °C and 80 °C were selected to zoom in near the Fermi level (Fig. 5b). At 30 °C, there are fewer electrons occupying the Fermi Energy *E*_F_, and the occupancy rises as a function of temperature up to 80 °C (Supporting Information Fig. S5). On the basis of crystal-field theory^53^,^54^, the chemical bonds are associated with the p-d hybridization between the O 2p and V 3d orbitals, and thus formed the d_‖_ and d_π_ orbitals (Fig. 5c). During the MIT, the lowering of the crystal symmetry from rutile to monoclinic lifts the orbital degeneracy. The formation of the V-V dimers along the rutile *c* axis splits the nonbonding d_‖_ states into occupied and unoccupied d_‖_^*^ states. Meanwhile the zigzagging of the V-V pairs shifts the d_π_ state to higher energies, which opens the band gap in the insulating phase^54^. The occupancy in the metallic state is higher than that in insulating state near the Fermi energy *E*_F_. This demonstrates that the electronic transition occurred during MIT for ultrathin VO_2_ films.

### DFT Calculations

**Figure 6:**
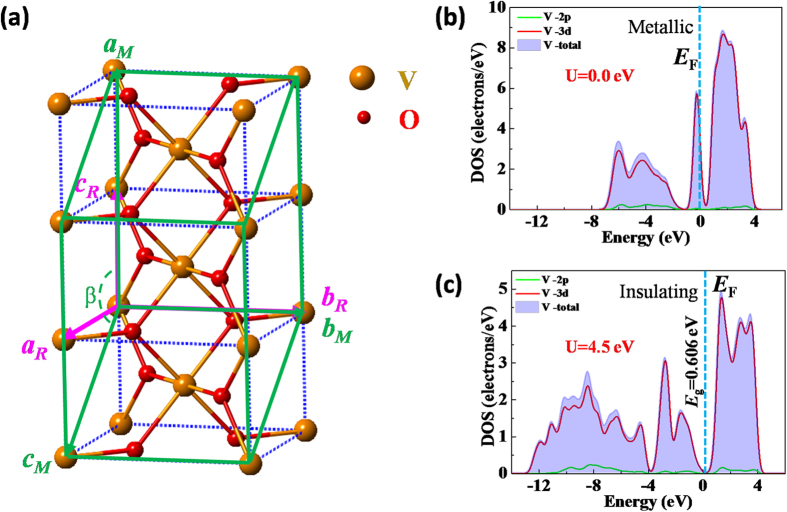
(**a**) The chosen super cell of VO_2_ for calculation. The green lines show the super cell of VO_2_ for DFT calculations. The calculated PDOS spectra of V with (**b**) U = 0.0 eV and (**c**) U = 4.5 eV.

We used density functional theory (DFT+U) calculations to verify the suppression of structural phase transition and modulation of the electron occupancy. The DFT+U method has well described the phase transition in VO_2_ system^55^,^56^, where U is the Hubbard electron−electron correlation parameter. Figure 6a shows the chosen super cell of VO_2_ for DFT calculations with different U. The experimental lattice constants under strained condition from our XRD results were used for calculations. For the tetragonal unit cell, the lattice constants are *a*_T_ = *b*_T_ = 4.584 Å, *c* = 2.831 Å, α_T_ = β_T_ = γ_T_ = 90°, and the lattice constants of super cell are *a*_s_ = 5.661 Å, *b*_s_ = 4.584 Å, *c*_s_ = 5.387 Å, α_S_ = γ_S_ = 90°, β_S_ = 121.7°, which are transformed from the tetragonal unit cell with the formula:


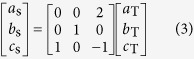


The calculated projected density of states (PDOS) for the metallic state is shown in Fig. 6(b) with Hubbard electron-electron correlation energy U = 0.0 eV. The PDOS for the insulating state with the chosen Hubbard electron-electron correlation energy U = 4.5 eV based on previous studies^55^,^57^,^58^ is plotted in Fig. 6c. (Please see the details of DFT calculations in Supporting Information Fig. S6)^55^,^59^. It is well understood that the high-temperature tetragonal phase of VO_2_ was metallic due to the electron occupancy at Fermi energy *E*_F_^57^—this is seen in Fig. 6b. However, the VO_2_ was an insulating state at low temperature because of the appearance of band gap *E*_g_ near the Fermi energy *E*_F_^57^, which may be induced by the electron-electron correlations. It is noted that the crystal structure used in calculations is locked whatever for the U = 0.0 eV and U = 4.5 eV. Therefore, we concluded that the ultrathin strained VO_2_ film changed from insulating to metallic states without the assistance of structural phase transition. The tetragonal insulating phase can survive with the help of epitaxial strain even at room temperature^58^. The electronic transition should be the only driving force for the MIT in this strained (001)-VO_2_/TiO_2_ epitaxial films.

### Thickness effect on the structural phase transition of strained VO_2_ films

Based on the above results and discussion, we can conclude that the structural phase transition during MIT for the ultrathin and nanoscale-strained (001)-VO_2_/TiO_2_ film is suppressed. The electronic transition and structural phase transition is separated in the highly strained (001)-VO_2_/TiO_2_ epitaxial films^26^. The electronic transition is the unique driving force that triggers the MIT. Therefore, the MIT of ultrathin VO_2_ films should be a pure Mott transition. This behavior can be ascribed to the interfacial epitaxial strain between the VO_2_ thin film and the TiO_2_ substrate^25^,^26^,^27^.

In terms of hetero-epitaxial systems^29^, the (001) oriented VO_2_ film is very easy to grow epitaxially along the lattice of tetragonal (001)-TiO_2_ substrate because of the tiny in-plane lattice mismatch (~−0.86%) between them^29^. Thus, the VO_2_ film is stabilized in the tetragonal structure at the initial interface under substrate constraint as described in the 13-nm-thick (001)-VO_2_/TiO_2_ epitaxial films. We also performed structural studies on the 24-nm-thick case. The results also evidenced the absence of the structural phase transition across the MIT (see Supporting Information Figs S7 and S8). Therefore, the entire ultrathin VO_2_ film may maintain tetragonal phase even at room temperature as long as the bi-axial strain in the (001)-VO_2_ thin film does not relax with the film-thickness below the critical thickness (~26.5 nm) in the hetero-epitaxial system.

According to the above discussion, the structural phase transition and electronic transition appeared simultaneously in the thicker VO_2_ films due to the relaxation of the strain. However, for the ultrathin strained VO_2_ films, the structural phase transition can be suppressed by the interfacial epitaxial strain in the vicinity of the MIT temperature. We conjecture that the structural phase transition may be finished at much lower temperature due to the strain effect, which will be studied in the future research.

## Conclusions

The (001)-VO_2_ films with various thicknesses were fabricated via RF magnetron sputtering, and the structural and electronic transitions were studied by temperature-dependent XRD-RSM, Raman spectroscopy, and UPS. The XRD-RSM results show that there is no structural phase transition during MIT, and the Raman spectra further confirms that the ultrathin VO_2_ film is tetragonal in both insulating and metallic states by epitaxial strain. The UPS confirmed that the MIT is induced solely by the electronic transition, which demonstrates that the MIT could be a pure Mott transition in the strained (001)-VO_2_/TiO_2_ epitaxial films. Furthermore, the absence of a structural phase transition is confirmed by DFT calculations. The electron-electron interactions should be the driving force for the MIT in the strained VO_2_ film. Our systematic study not only provide a better understanding of the MIT mechanism for strained (001)-VO_2_/TiO_2_ thin films, but also show some clues to modulate the MIT via strain engineering. Furthermore, a series thickness of (001)-VO_2_/TiO_2_ around the critical thickness (~26.5 nm) should be prepared for further confirmation the strain-controlled behaviors of the MIT. Moreover, much more experimental and theoretical studies for the MIT mechanism of strained or relaxed VO_2_ films on the other oriented TiO_2_ ((011) or (111)) substrates are desired in future, which will be helpful to understand the MIT mechanism of the strained VO_2_/TiO_2_thin films comprehensively.

## Methods

### Sample fabrication

The ultrathin VO_2_/TiO_2_ films were fabricated on (001)-oriented TiO_2_ substrates purchased from Hefei Kejing Materials Technology Corporation using reactive radio frequency (RF) magnetron sputtering. A 99.99%-purity vanadium metal target was sputtered at 60 W RF power at a sputtering pressure of 0.43 Pa. The growth temperature was set to 400 °C and the Ar:O_2_ flow ratio was 60:0.5^60^.

### X-ray diffraction characterization

To characterize the growth quality and structure of the VO_2_ films, both conventional XRD scans and synchrotron radiation reciprocal space mapping (RSM) were performed. The XRD *θ*–2*θ* and *φ* scans were performed on a four-circle diffractometer with a Ge (220) × 2 incident-beam monochromator (Rigaku SmartLab Film Version, Cu-*Kα* radiation). The RSMs were collected at beamline BL14B1 on the Shanghai Synchrotron Radiation Facility (SSRF, *λ* = 0.124 nm). The results were plotted in the reciprocal lattice units (r.l.u.) of TiO_2_.

### Raman spectroscopy

*In situ* temperature-controlled Raman spectroscopy with acquisition time of 20 s was acquired using the XploRA^TM^ Raman spectrometer (HORIBA Scientific, Ltd). A 532 nm laser of 0.25 mW was used as the excitation source with a 100× microscope objective. To perform temperature-dependent measurements, a home-made, actively-controlled heating stage was equipped on the sample with an accuracy of 0.5 °C. Each Raman spectrum was acquired three times after the temperature was stable.

### Ultraviolet Photoelectron Spectroscopy

Ultraviolet Photoelectron Spectroscopy (UPS) was used to study the V 3d electronic states near the Fermi energy *E*_F_. These experiments were performed at the Catalysis and Surface Physics Endstation with the BL11U beamline on the National Synchrotron Radiation Laboratory (NSRL) in Hefei, China. The temperature-dependent valence-band spectra were measured with a VG Scienta R4000 analyzer with photon energy of 21.2 eV as the excitation source—this was calibrated with the Fermi level determined by the Au sample. In addition, the heating controller (temperature control range: 90–1400 K) offers a stable and precise temperature with 0.1 °C accuracy. Prior to UPS measurements, the films were subjected to argon ion sputtering to expose a clean surface of the film to UV light.

### Theoretical calculations

The density functional theory (DFT) calculations were carried out with DFT+U where U is the Hubbard electron-electron correlation parameter. We used the Materials Studio calculation package equipped on Supercomputing Center of the University of Science and Technology of China. We adopted the Perdew-Burke-Ernzerhof functional under generalized gradient approximation (GGA) for the exchange correlation along with double-ζ-double polarized basis set for the electron wave function.

## Additional Information

**How to cite this article**: Yang, M. *et al*. Suppression of Structural Phase Transition in VO_2_ by Epitaxial Strain in Vicinity of Metal-insulator Transition. *Sci. Rep.* **6**, 23119; doi: 10.1038/srep23119 (2016).

## Supplementary Material

Supplementary Information
