# Supplementary material for: Suppression of Structural Phase Transition in VO2 by Epitaxial Strain in Vicinity of Metal-insulator Transition
Source: Sci Rep. 2016 Mar 15;6:23119. doi: 10.1038/srep23119 (PMC4792152; doi:10.1038/srep23119)
Supplement: Supplementary Information [file srep23119-s1.pdf]

Supporting Information

Suppression of Structural Phase Transition in VO_2 by Epitaxial Strain in Vicinity of Metal-insulator Transition

Mengmeng Yang[a,b], Yuanjun Yang[a,b,c*], Bin Hong[a,b], Liangxin Wang[a], Kai Hu[a], Yongqi Dong[a,b], Han Xu[d], Haoliang Huang[c], Jiangtao Zhao[a], Haiping Chen[a], Li Song[a], Huanxin Ju[a], Junfa Zhu[a], Jun Bao[a,b,c], Xiaoguang Li[d], Yueliang Gu[e], Tieying Yang[e], Xingyu Gao[e], Zhenlin Luo[a,b*], Chen Gao[a,b,c*]

[a] *National Synchrotron Radiation Laboratory, University of Science and Technology of China, Hefei, Anhui 230026, China*

[b] *Collaborative Innovation Center of Chemistry for Energy Materials, University of Science and Technology of China, Hefei, Anhui 230026, China*

[c] *CAS Key Laboratory of Materials for Energy Conversion, Department of Materials Science and Engineering, University of Science and Technology of China, Hefei, Anhui 230026, China*

[d] *Hefei National Laboratory for Physical Sciences at the Microscale and Department of Physics, University of Science and Technology of China, Hefei, Anhui 230026, China*

[e] *Shanghai Synchrotron Radiation Facility, Shanghai Institute of Applied Physics, Chinese Academy of Sciences, Shanghai 201204, China*

*Author to whom correspondence should be addressed. Electronic mail: 1) yangyuanjun@ustc.edu.cn, 2) cgao@ustc.edu.cn or 3) zlluo@ustc.edu.cn.

1. The temperature-dependent XRD θ-2θ scans of 60 nm (001)-VO_2/TiO_2

Figure S1 shows the temperature-dependent XRD θ-2θ scans of 60 nm (001)-VO_2/TiO_2 and the obvious peak shifts at low (~30 ^{o}C) and high temperature (~90 ^{o}C) are observed. Based on this result, the lattice constant *c* was calculated and shown in Figure 3 in the main text. The lattice constant *c* has a sharp jump across the MIT, indicating an existing structural phase transition in the thicker films.

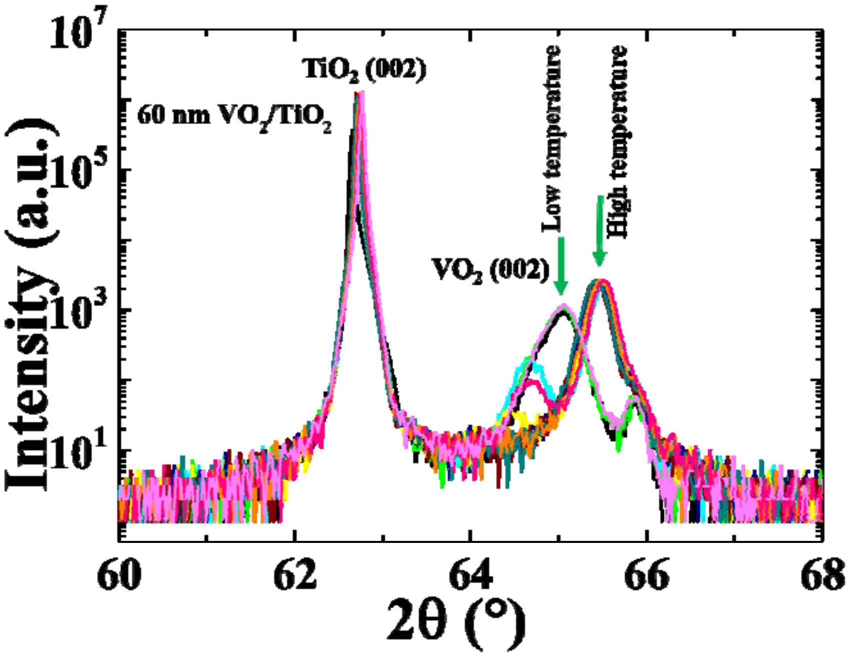

Figure S1 The temperature-dependent XRD θ-2θ scans of 60 nm (001)-VO_2/TiO_2.

2. The R-T curves of 60 nm (001)-VO_2/TiO_2

Figure S2 shows the R-T curve and its differential curve of 60 nm (001)-VO_2/TiO_2. It can be seen from Figure S2 that the resistance jumps sharply about four orders at about 65 °C across the MIT, which indicates the high quality of the VO_2 film grown on the TiO_2 substrate by magnetron sputtering techniques. From the inset of differential curve, the MIT ranges from 50 °C to 65 °C, which was also labeled in Figure 3 in the manuscript.

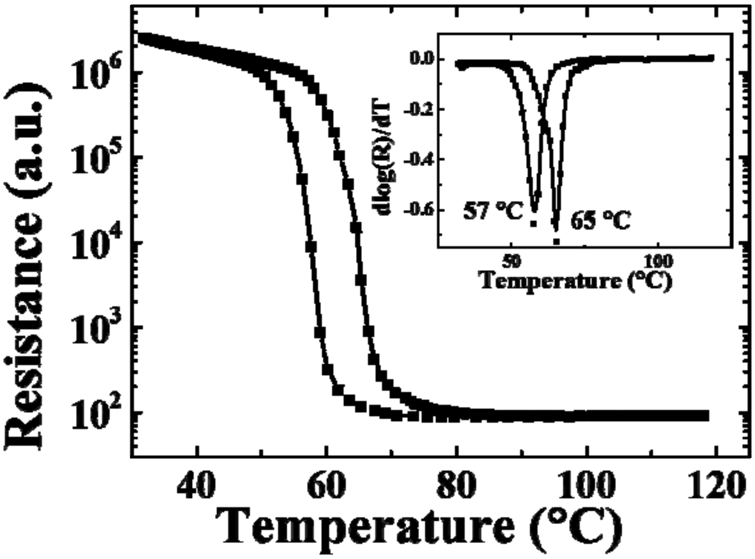

Figure S2 The R-T curve and its differential curve of 60 nm (001)-VO_2/TiO_2.

3. (001)-VO_2/TiO_2 epitaxial films both at heating and cooling process.

Figure S3 shows the complete temperature-dependent Raman spectrum of 13-nm-thick (001)-VO_2/TiO_2 epitaxial film both at heating and cooling process. There are no strong sharp peaks belong to the monoclinic VO_2 phase and the 358 cm^{-1} and 413 cm^{-1} Raman peaks belong to tetragonal VO_2 phase are existed both at low and high temperature. Furthermore, the positions and intensities of them are not changed anymore, which suggests that the structural phase transition should be absent across the MIT both at heating and cooling process.

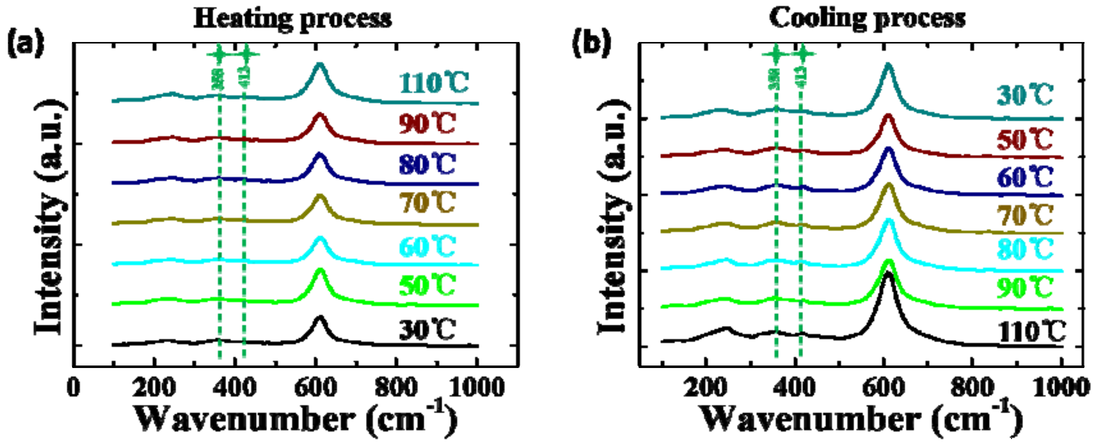

Figure S3 The complete temperature-dependent Raman spectrum of (001)-VO_2/TiO_2 epitaxial films in 13-nm thick both at (a) heating and (b) cooling process.

4. The temperature-dependent Raman spectrum of bare TiO_2 substrate.

For comparison, Figure S4 shows the temperature-dependent Raman spectrum of bare (001)-TiO_2 substrate and the characteristic peaks (140 cm^{-1}, 242 cm^{-1}, 446 cm^{-1} and 609 cm^{-1}) are labeled. It can be seen that the Raman peaks are not changed no matter for the positions or intensities. This result is useful for eliminating the substrate effect on the Raman peaks for the (001)-VO_2/TiO_2 epitaxial thin films.

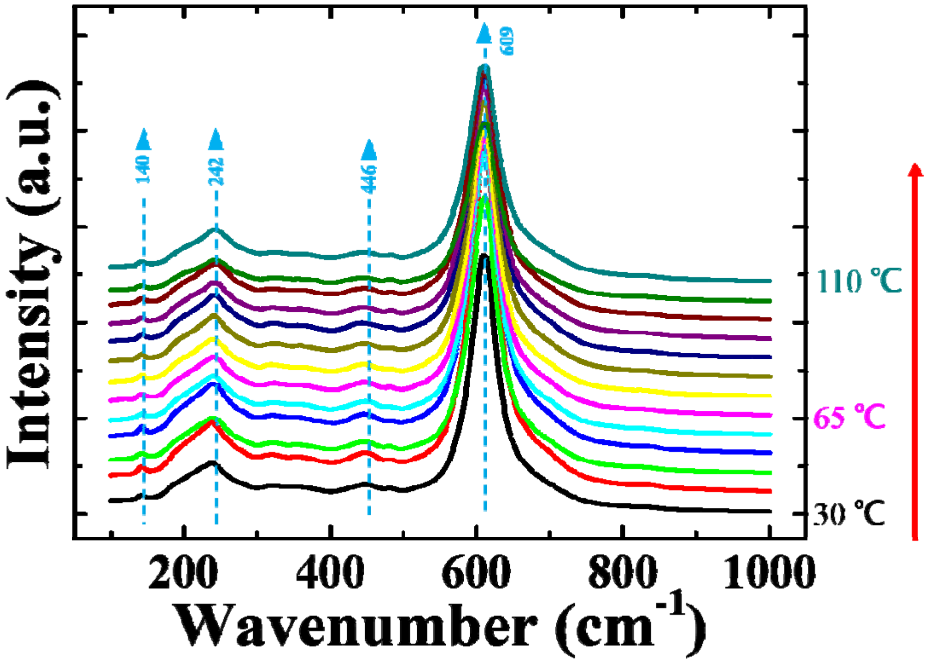

Figure S4 The temperature-dependent Raman spectrum of bare TiO_2 substrate.

5. The normalized intensities of V 3d electron state at Fermi energy E_F as a function of temperature.

Figure S5 shows the temperature-dependent intensities of V 3d electron state at Fermi energy E_F. The intensities were normalized to the integrated intensity from Binding energy from -0.2 to 0.5 eV as shown in Figure 5b in our manuscript. The intensities shows a change across MIT, which implies that the VO_2 should undergo an electronic phase transition across the MIT, which was well consistent with the previous studies (see references in the main text). This may further verifies that the electronic phase transition should lead to the occurrence of the MIT of the strained 13-nm (001)-VO_2/TiO_2 epitaxial thin films.

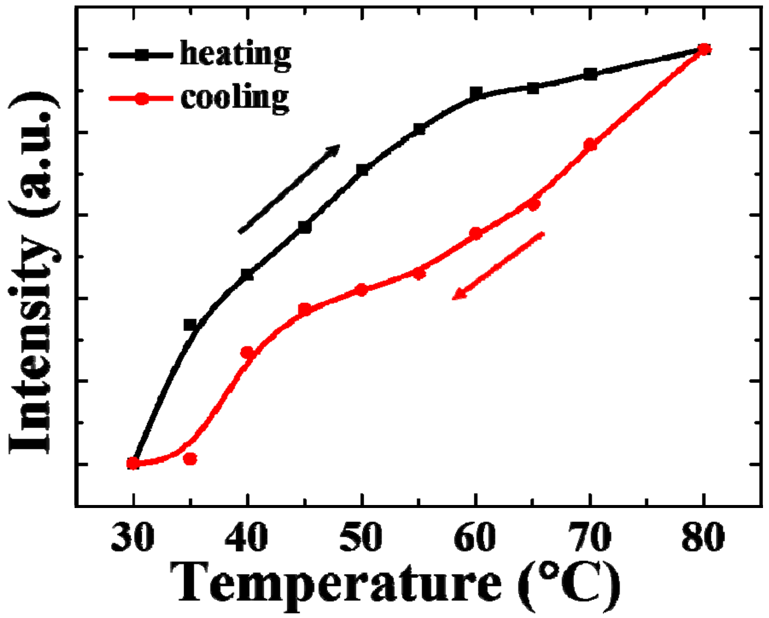

Figure S5 The normalized intensities of V 3d electron state at Fermi energy E_F as a function of temperature.

6. The complete calculated density of state (DOS) spectra of VO_2 with different Hubbard electron-electron correlation energy.

We adopted DFT+U method to study the DOS spectra in the 13-nm VO_2 film in the present work. The crystal structure was determined by our experiments and did not change for the cases of U=0.0 eV and U=4.5 eV. This assumption was intended to show the dominant role of electron-electron correlation. Here, we adopted the generalized gradient approximation (GGA) for the exchange correlation along with double-ζ-double polarized basis set for the electron wave function. The computed projected density of states (PDOS) is shown in Figure S6. It is seen that the DOS spectra with U=0.0 eV that the VO_2 was metallic due to the electron occupancy at Fermi energy E_F in Figure S6 (a). On the other hand, the DOS spectra with U=4.5 eV in Figure S6 (b) demonstrated the VO_2 was insulating state because there was a band gap of 0.606 eV near Fermi energy E_F. The band gap (~0.606 eV) with U=4.5 eV, agrees well with the orbital distributions theoretically calculated by Quackenbush *et al.* [Nano Lett. 2013, 13, 4857.] and Gabriel Kotliar *et al.* [Phys. Rev. B 2010, 81, 115117.] Moreover, the open band gap is in excellent agreement with the experimental results of 0.6 V. [Rev. B 2013, 87, 115121. Phys. Rev. B 2013, 87, 195106.] Therefore, with the addition of electron-electron correlation interaction U term (~4.5 eV), the V d-orbitals split into a lower band and an upper band and eventually produce a band gap without changing the crystal structure. Consequently, the electron-electron correlation may induce the MIT, where the structural phase transition is not the mandatory requirement for the MIT.

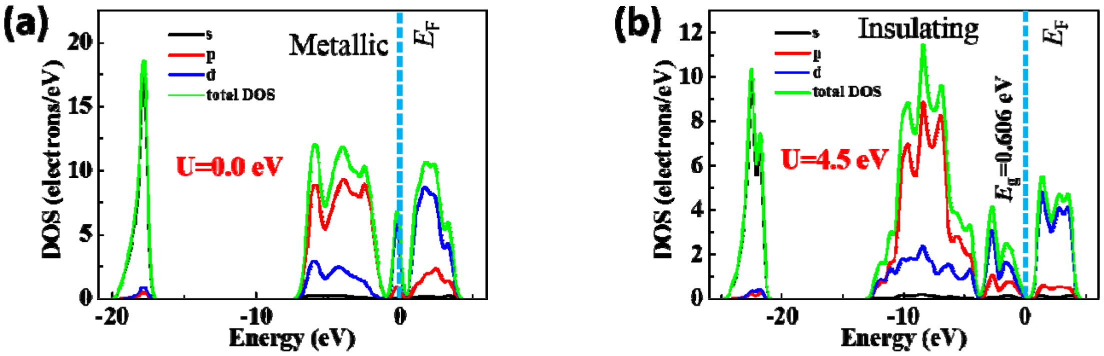

Figure S6 The calculated DOS spectra of with (a) U=0.0 eV and (b) U=4.5 eV.

7. The temperature-dependent XRD of the 24-nm-thick VO_2/TiO_2 film.

Figure S7(a) and (b) shows the temperature-dependent XRD θ-2θ scans of 24-nm (001)-VO_2/TiO_2 and there is no obvious peak shifts during both heating and cooling process. Moreover, in Figure S7(c), the lattice constant *c* has no jumping behavior and has a linear relationship with respect to the temperature. These results indicate that there is also no structural phase transition in the 24-nm-thick (001)-VO_2/TiO_2 film. Comparing the XRD results of the 13-nm and 60-nm VO_2 films, we can conclude that the VO_2 films thinner than the critical thickness (~26.5 nm) may have no structural phase transition across the MIT.

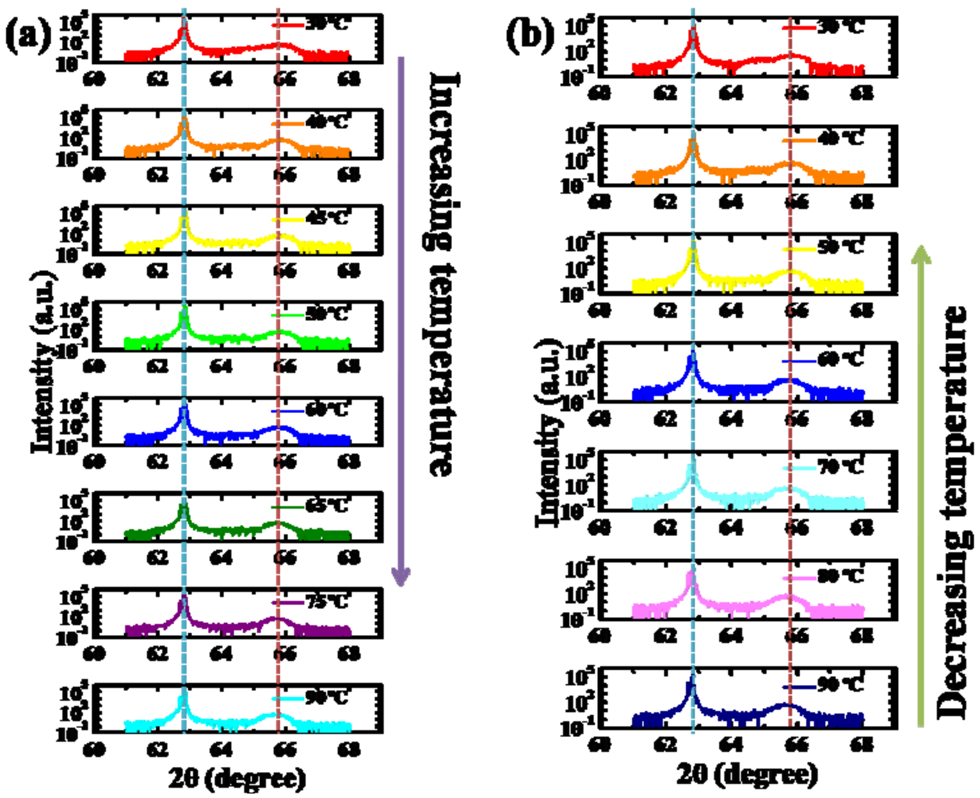

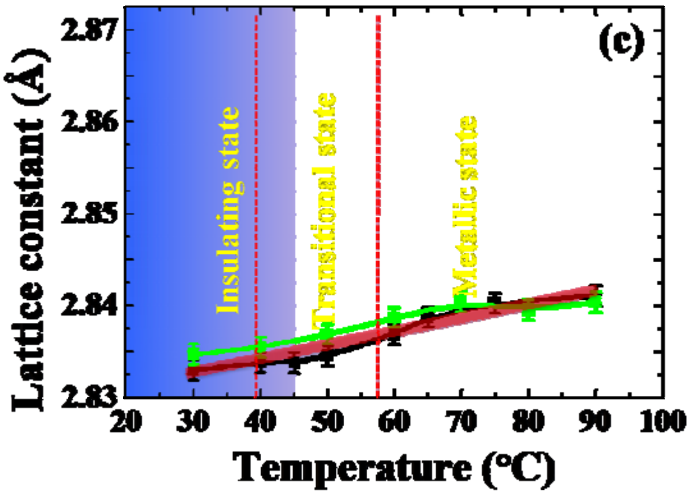

Figure S7 The temperature-dependent XRD θ-2θ scans of (002) peaks of the 24-nm-thick VO_2/TiO_2 film. The heating and cooling processes are shown in (a) and (b), respectively. (c) The lattice constant *c* is as function of the temperature. The dotted lines show that region of the MIT.

8. The temperature-dependent Raman spectrum of the 24-nm-thick VO_2/TiO_2 film.

Figure S8 shows the complete temperature-dependent Raman spectrum of the 24-nm-thick (001)-VO_2/TiO_2 epitaxial film during MIT. There are no strong sharp

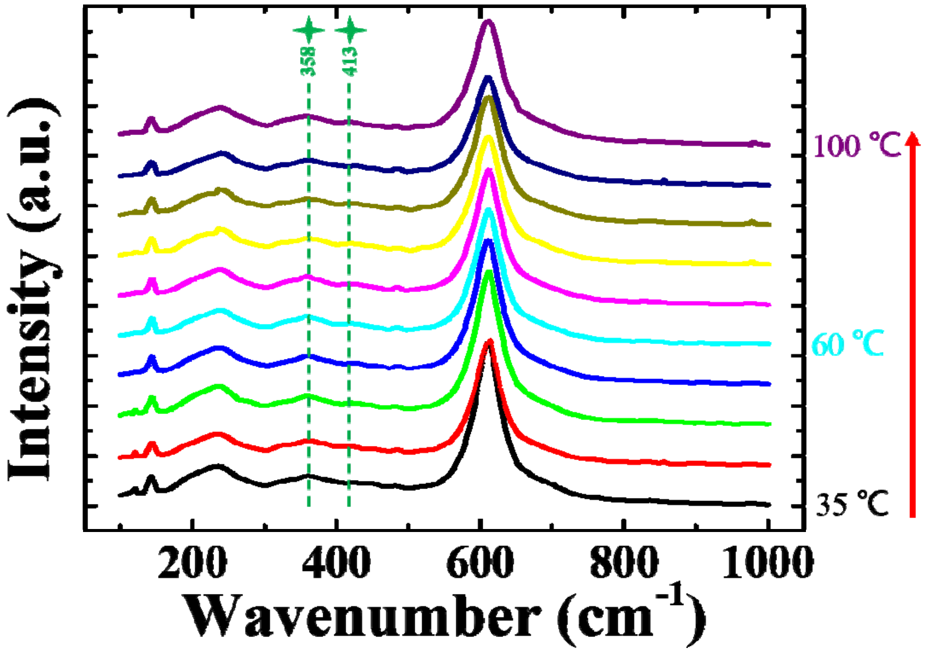

Figure S8 The temperature-dependent Raman spectrum of the 24-nm-thick VO_2/TiO_2 film.

peaks belong to the monoclinic VO_2 phase and the 358 cm^{-1} and 413 cm^{-1} Raman peaks belong to tetragonal VO_2 phase are existed both at low and high temperature. This suggests that the structural phase transition should be absent across the MIT. Comparing the Raman spectrum of the 13-nm and 60-nm VO_2 films, we can conclude that the VO_2 films thinner than the critical thickness (~26.5 nm) maintain tetragonal phase even at room temperature by epitaxial strain.
